# Rail induced lateral migration of particles across intact co-flowing liquids

**DOI:** 10.1038/s41598-022-26387-5

**Published:** 2022-12-16

**Authors:** Iwona Ziemecka, Amaury de Hemptinne, Vyacheslav R. Misko, Matthieu Briet, Pierre Gelin, Ilyesse Bihi, Dominique Maes, Wim De Malsche

**Affiliations:** 1 µFlow Group, Department of Chemical Engineering, Vrije Universiteit Brussel, Pleinlaan 2, 1050 Brussels, Belgium; 2 Structural Biology Brussels, Vrije Universiteit Brussel, Pleinlaan 2, 1050 Brussels, Belgium

**Keywords:** Chemical engineering, Biomedical engineering, Fluids, Fluidics, Surface patterning, Fluid dynamics

## Abstract

This paper presents a rail guided method to apply a Layer-by-Layer (LbL) coating on particles in a microfluidic device. The passive microfluidic approach allows handling suspensions of particles to be coated in the system. The trajectory of the particles is controlled using engraved rails, inducing lateral movement of particles while keeping the axially oriented liquid flow (and the interface of different liquids) undisturbed. The depth and angle of the rails together with the liquid velocity were studied to determine a workable geometry of the device. A discontinuous LbL coating procedure was converted into one continuous process, demonstrating that the chip can perform seven consecutive steps normally conducted in batch operation, further easily extendable to larger cycle numbers. Coating of the particles with two bilayers was confirmed by fluorescence microscopy.

## Introduction

The ability to manipulate microparticles is crucial for many applications in engineering, chemistry, biology, and physics. Various applications require particle processing, sorting, or self-assembly. Designing advanced particles requires the use of deposition processes in order to produce complex, nanostructured building blocks. One of the deposition techniques that is very popular nowadays is the Layer-by-Layer assembly (LbL) method ^1,2^ introduced by Decher et al. This method has many advantages: its simple preparation, versatility, enhancement of material properties, control over material structure, porosity, robustness, possibility of applying high biomolecules loads in the films^3^. The LbL method received considerable attention in engineering and biomedical fields and is applied for example in drug delivery, integrated optics, sensors and friction reducing coatings. In the classical LbL method, thin films are formed by subsequent deposition of oppositely charged polyelectrolytes (polymer electrolytes) on a substrate of any shape, resulting in polyelectrolyte multilayers. Adsorption of the film is mainly the result of electrostatic interactions occurring between polycationic and polyanionic electrolytes. The layer can be achieved in multiple ways, for example, by dip-coating, spin-coating or spray-coating. Automation of the LbL processes using conventional macro-scale reactors is highly desirable but difficult to implement. These time-consuming and non-continuous processes generally require bulky and expensive equipment. Moreover, problems as non-uniformity and aggregation of microcapsules are often encountered, requiring the application of downstream processing steps like centrifugation, washing and re-suspension. Also, consumption of reagents is higher in batch processes, which can be an important factor when e.g. an expensive drug is involved.

Handling of particles is essential in particle manufacturing approaches. Among many available techniques, optical tweezers are remarkably powerful to manipulate individual objects. Optical tweezers use forces exerted by a strongly focused beam of light to trap and move particles ranging in size from tens of nanometers to tens of micrometers and can be used to organize planar assemblies of colloidal particles, but also to construct optical pumps and valves built of colloidal particles in microfluidic channels activated with optical tweezers^4–6^. Another technique to manipulate particles uses sound waves, requiring a lower power density than optical tweezers. Ding et al. developed an acoustic device, based on standing surface acoustic waves that can trap and manipulate single microparticles with real-time control^7^. A continuous flow acoustic standing wave is used for the separation of particles in a size range of tens of nanometers to tens of micrometers. Acoustic tweezer technology facilitates particle focusing, separation, alignment, and patterning^8–10^. A focused surface acoustic wave (FSAW) was used in a microfluidic setting to produce microcapsules with a core–shell structure^11^. Magnetic particles can be manipulated in microfluidic channels with the use of magnetic field^12,13^. Magnetism has been used in microfluidics for actuation, manipulation, and detection. The forces involved in micro-magnetofluidics have been extensively described and are generally well understood^14^. Many applications have been developed so far, with as a prominent example continuous flow magnetic separation of particles and cells^15^. Another active method to control particles motion is tilted-angle dielectrophoresis^16^. The zig-zag trajectory of particles through three parallel laminar streams was realized by means of pairs of adjacent tilted parallel electrodes arranged in a zigzag fashion around the microfluidic channel^16^. Methods to control the motion of microparticles in microfluidic devices have already been extensively studied and reported^17^.

The above-described technologies, based on optics, acoustics, magnetism or dielectrophoresis demand additional, external forces and sometimes apply only to the particles of specific (e.g., magnetic) properties. Moreover, some of those methods require very expensive equipment.

Passive control of the motion of microparticles in microfluidics is challenging but has been performed successfully. There are methods relying on inertial effects or on guiding structures, with the channel and functional structure design as the critical element that enables particle manipulation.

Inertial microfluidics uses fluid inertia for enhancing mixing and inducing particle separation and focusing^18^. By integration of curved channels (e.g., spiral), inertial microfluidics can be used for continuous separation of particles based on their size^19^. Sangupta et al. noticed that colloid particles can follow lines (grooves) in microfluidic chip^20^. These defect lines were random and trajectory lines were not deliberately designed. Others focused on controlling the trajectory of the particles in microfluidic chip using designed guiding structures. Park et al. studied the ability to sort tailored particles that fit the rail only if they have a specific orientation^21^. The concept of rails for specially designed particles was also used not only to guide them but also to assemble them on chip^22–24^.

More diversity in guiding structures can be found when droplets are to be manipulated. Kantak et al. imposed droplet trajectories by obstacles to coat droplets LbL on chip^25^. Another method was developed as well, with the droplets confined by a channel roof and trapped and guided by rails and anchors that were etched in the channel top surface. To reduce the surface energy of droplets, they enter a local depression^26^. Ahn et al. presented a simple method of guiding, distributing, and storing of a train of droplets by using side flows, cavity guiding tracks and storage chambers^27^. Rail structures were also used for sorting of gas bubbles in liquid^28^.

Another method involves magnetic interaction. Ferromagnetic rails are used to locally create magnetic potential wells. When the field is turned off, the magnetic droplets follow the liquid flow. By switching on the magnetic field, droplets experience a magnetic force that affects their trajectory when passing over the magnetized rail^29^. A combination of active laser (optical) manipulation and passive manipulation by the structures like rails and anchors was used in microfluidics to pattern 2D arrays with droplets in a highly selective manner^30^.

In the present paper, we present a concept to manipulate particles trajectory in microfluidic channels. We introduce a passive method that does not require an external force or specific properties of the microparticles. We show the use of a particle guiding rail with the aim to contact the particle with multiple liquids running in parallel. The impact of the rail geometry (height and the angle of the rail) on the stability of the particle guided motion as well as of the (interface) of the co-flow liquids is studied. Using rails at the bottom of the channel is more practical than using structures in the microfluidic channel (like pillars) because it is less prone to clogging during LbL coating, which has an impact on assuring the stability of multilaminar flows.

We then construct an operational map of obtained liquid regimes and apply this knowledge to design and validate a microfluidic chip that replaces seven consecutives steps in a LbL coating methodology. The on-chip coating is fast, requiring only a few tens of seconds.

## Results and discussion

### Chips and liquids

#### Chip

**Figure 1:**
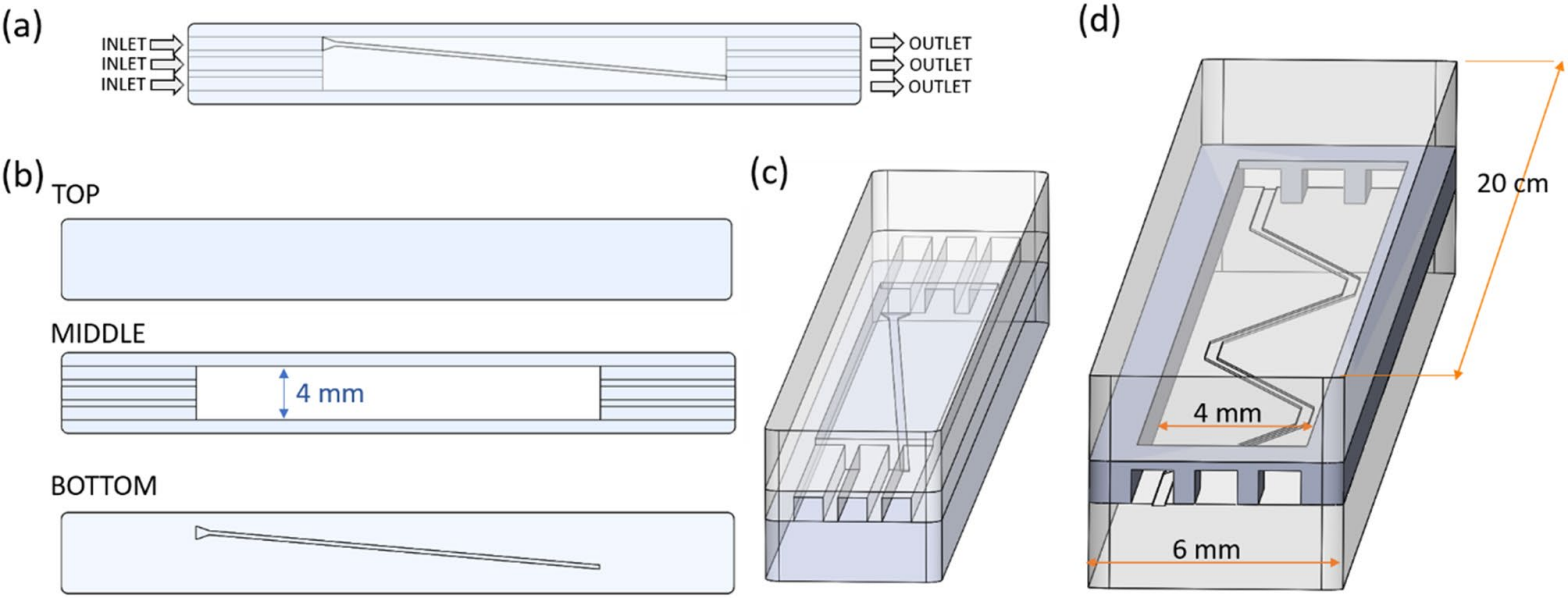
Scheme of the chip: (**a**) top view, (**b**) parts of the chip, (**c**) side view, (**d**) a zig-zag chip [not to scale: the zig-zag chip is longer than a single-rail chip in (**c**) (50 mm)].

**Figure 2:**
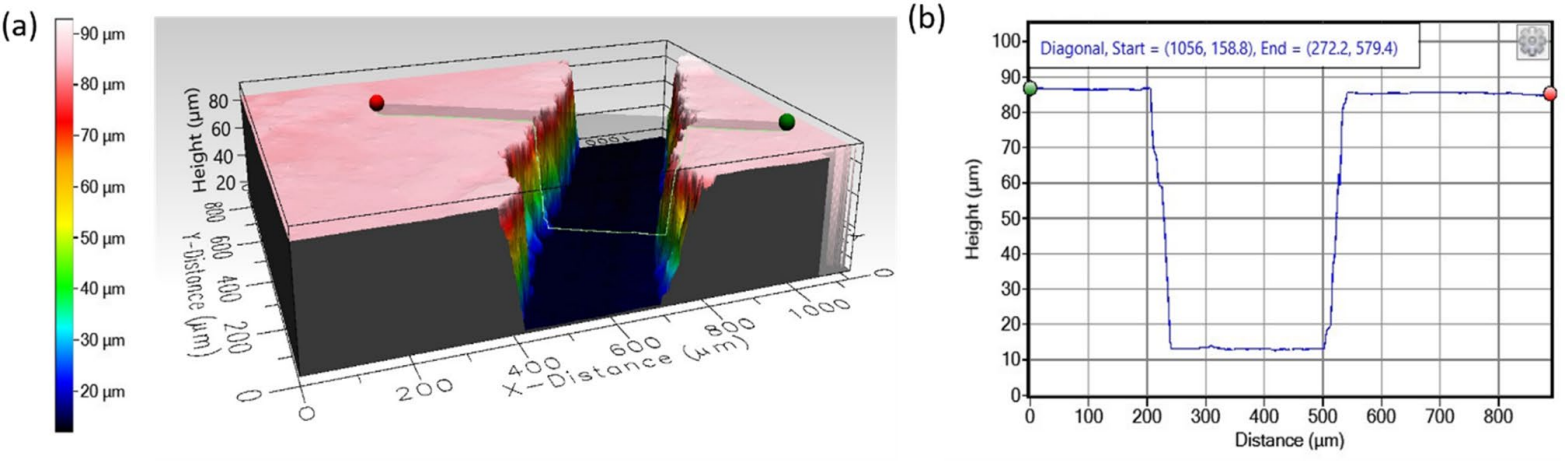
Profilometer measurement of the rail depth before bonding the parts of the chip: (**a**) 3D view, (**b**) line profile.

The chip was fabricated in-house by milling polymethyl-methacrylate (PMMA) as substrate. The chip outline is presented in Fig. 1. The chip is composed of three different layers that were subsequently assembled and bonded. The chip has three inlets and three outlets and the dimensions of the top layer are 6 mm × 50 mm (2 mm thick). The middle layer is 1 mm thick and has a 4 mm wide and 30 mm long channel. The bottom part of the chip is 2 mm thick and has a groove (rail) milled on its surface. At the beginning of the rail there is a groove area in the shape of a triangle to facilitate entrapment of the particles introduced with the liquid during the experiment. The width of the rail is 300 µm and the depth of the rail is 45 to 310 µm, depending on the chip design. The depth of the rail was determined by a profilometer (Filmetrics Profilm 3D). Measurements were performed at five different positions for each rail (Fig. 2) before bonding the chip. The same method was applied to measure the roughness, *R*_g_, of the bottom surface of the chip and the rail. After testing different angles of rails: 0°, 5°, 10°, 15°; a zig-zag chip was designed and fabricated (Fig. 1d). The zig-zag chip was designed for on-chip LbL coating of particles and is built, similarly to the previous chips, from three layers, but it is longer (the top layer has dimensions 6 mm × 20 cm, and the middle layer has a 4 mm wide and 18 cm long channel) and its rail is built of 0° and 5° rails connected together in the shape of a zig-zag. The zig-zag chip can therefore accommodate four rail segments at 5° with respect to the channel direction (see Fig. 1d). As an example of a zig-zag chip, an image of a PMMA chip with zig-zag step channel is shown in Fig. 1S.

Ethanol was chosen as carrier liquid to study the behavior of the liquid flow in the chip for multiple reasons, with the most important one that it is an excellent solvent for many chemicals. Moreover, it wets PMMA which ensures easy removal of gas bubbles and prevents particles to stick to the surface.

**Figure 3:**
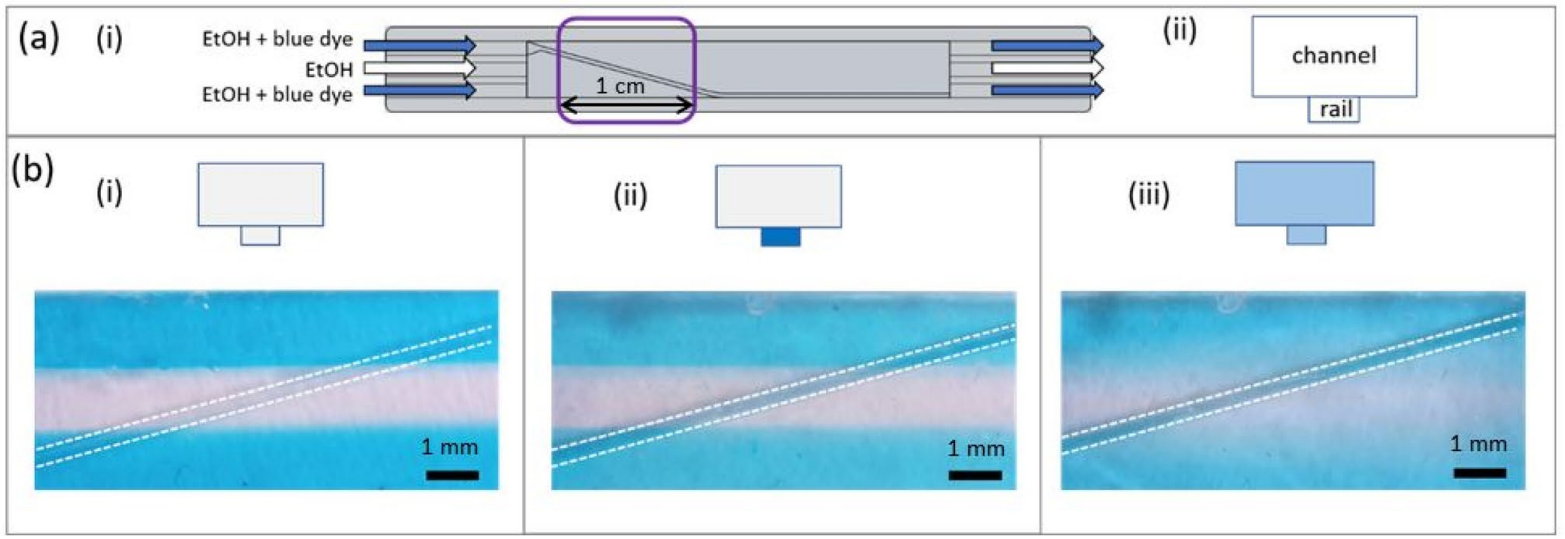
(**a**) Scheme of the experiment: (i) top view of the chip, (ii) cross section of the chip. (**b**) Three regimes observed for a rail at an angle of 15°, scale bar 1 mm.

Ethanol was introduced to the chip through the three inlets. The middle stream was pure while the adjacent streams were colored with blue dye to visualize the flow, see Fig. 3a. The flow rate of all three liquids was controlled with a syringe pump. The liquids were always introduced at the same flow rate, ranging from 20 to 240 mL h^−1^ with corresponding linear liquid velocities of 4.2 to 50 mm s^−1^. Note that these values refer to average liquid velocities in the chip. The liquid velocity is maximal at the central part of the flow and decreases to zero at the boundary. Therefore, at the level of particles moving near the bottom of the chip the fluid velocity is substantially smaller than the average value. The actual fluid velocities at the level of particles are estimated below in “Methods”, “Fluid velocity profile”.

When performing the experiments, three types of behavior of liquid flow were observed, further referred to as regimes 1, 2 and 3, see Fig. 3b.

#### Regime 1

Three clear stripes in blue-transparent-blue co-flowing liquids are observed. The liquid in the entire area of the middle stream is transparent. This means that (transparent) ethanol is in the rail and in the channel above the rail. The rail is filled by the different liquids depending on the position of the rail in the chip. It is filled by the liquid that flows above it.

#### Regime 2

Although the co-flow of the three liquids can be distinguished, blue colored ethanol is present along the entire length of the rail. This is visible in the chip where transparent ethanol flows in the middle of the channel while below blue colored ethanol flows inside the rail. The beginning of the rail is at the entrance of the chip where blue colored ethanol is introduced. This liquid invades the rail and fills it up through all its length.

#### Regime 3

Clear borders between co-flowing liquids are no longer observed. Blue dye covers the area of the middle stream.

**Figure 4:**
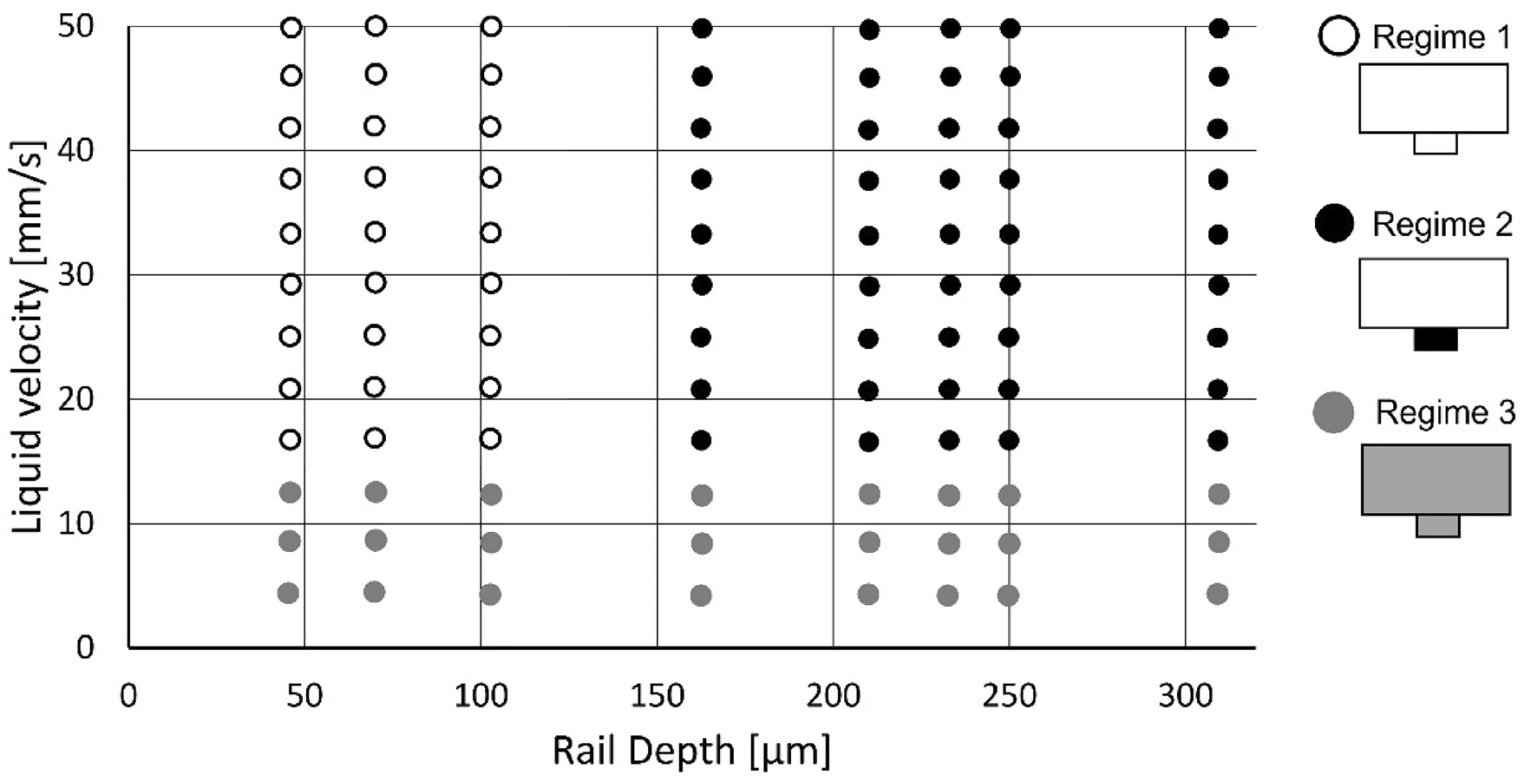
Map showing where the three regimes of liquid transport in a rail are observed as a function of rail depth and liquid flow rate.

The occurrence of the three regimes of liquid transport in the rail as a function of rail depth and liquid velocity is summarized in Fig. 4. Regime 1 was observed for velocities of the liquids > 15 mm s^−1^ with a rail depth < 100 µm. Regime 2 was observed for velocities of the liquids > 15 mm s^−1^ and rails of the depth > 160 µm. Regime 3 was observed for low velocities of the liquids < 15 mm s^−1^ for all tested rails.

With the aim to provide conditions for repetitive chemical treatments on particles, only the conditions leading to Regime 1 are considered appropriate because each particle traveling in the rail must be in contact with the liquid of the same nature as the liquid above the rail. For LbL coating procedures, e.g., particles must travel through three different liquids to undergo bilayer coating, i.e.: liquid with first coating agent, then rinsing liquid, then liquid with second coating agent.

### Particles on rail

**Figure 5:**
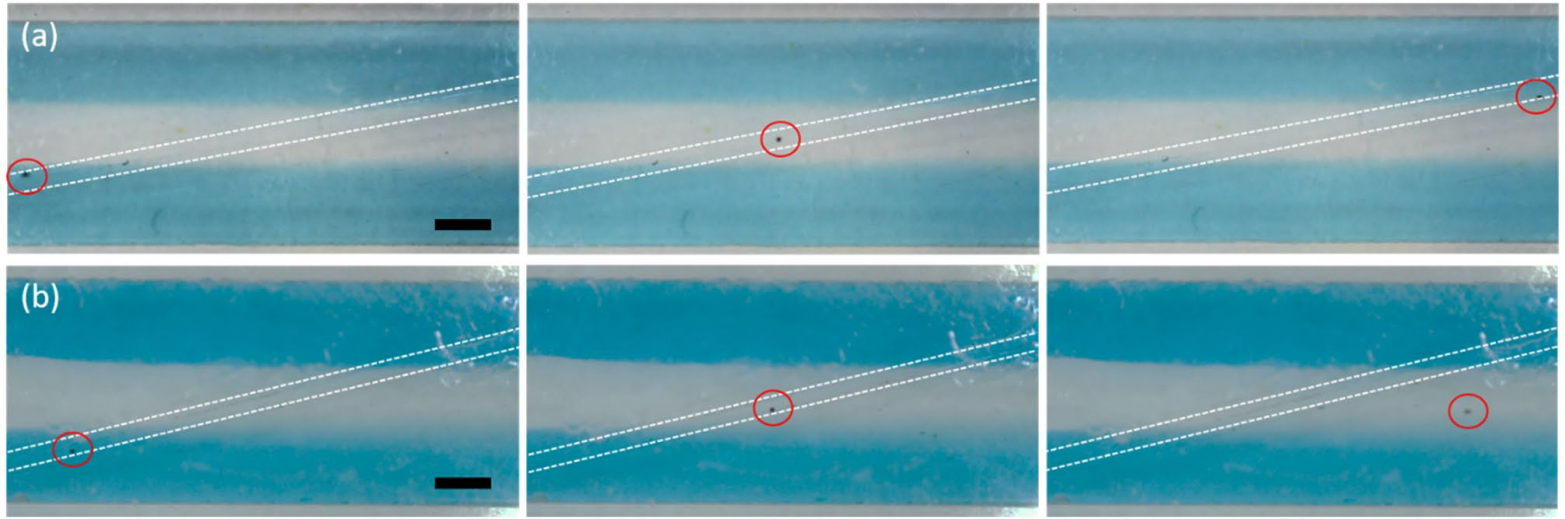
(**a**) Particle following the rail of 10°, depth 45 µm, liquid velocity 12.5 mm/s; (**b**) particle escaping the rail of 15°, depth 73 µm, liquid velocity 12.5 mm/s. Scale bars are 1 mm.

In order to test how particles follow the rail, spheres of 89 µm diameter were introduced in the chip as a suspension in blue colored ethanol. The suspension was introduced at the inlet, which is connected to the initial part of the rail. The velocity of the particles introduced to the chip quickly decreases as soon as the particles touch the bottom of the chip. It is crucial that the particles touch the surface of the bottom of the chip to be able to remain inside the rail. The area of the chip (4 mm wide × 10 mm long) where the rail crossed the liquid flow was monitored to evaluate whether the particles follow the rail, therewith crossing three streams of liquid (see Fig. 5). The particle must follow the rail without colliding with other particles. If they do, this can lead to bumping and subsequent escape of one or both particles. Only single particle events were considered in the present study.

**Figure 6:**
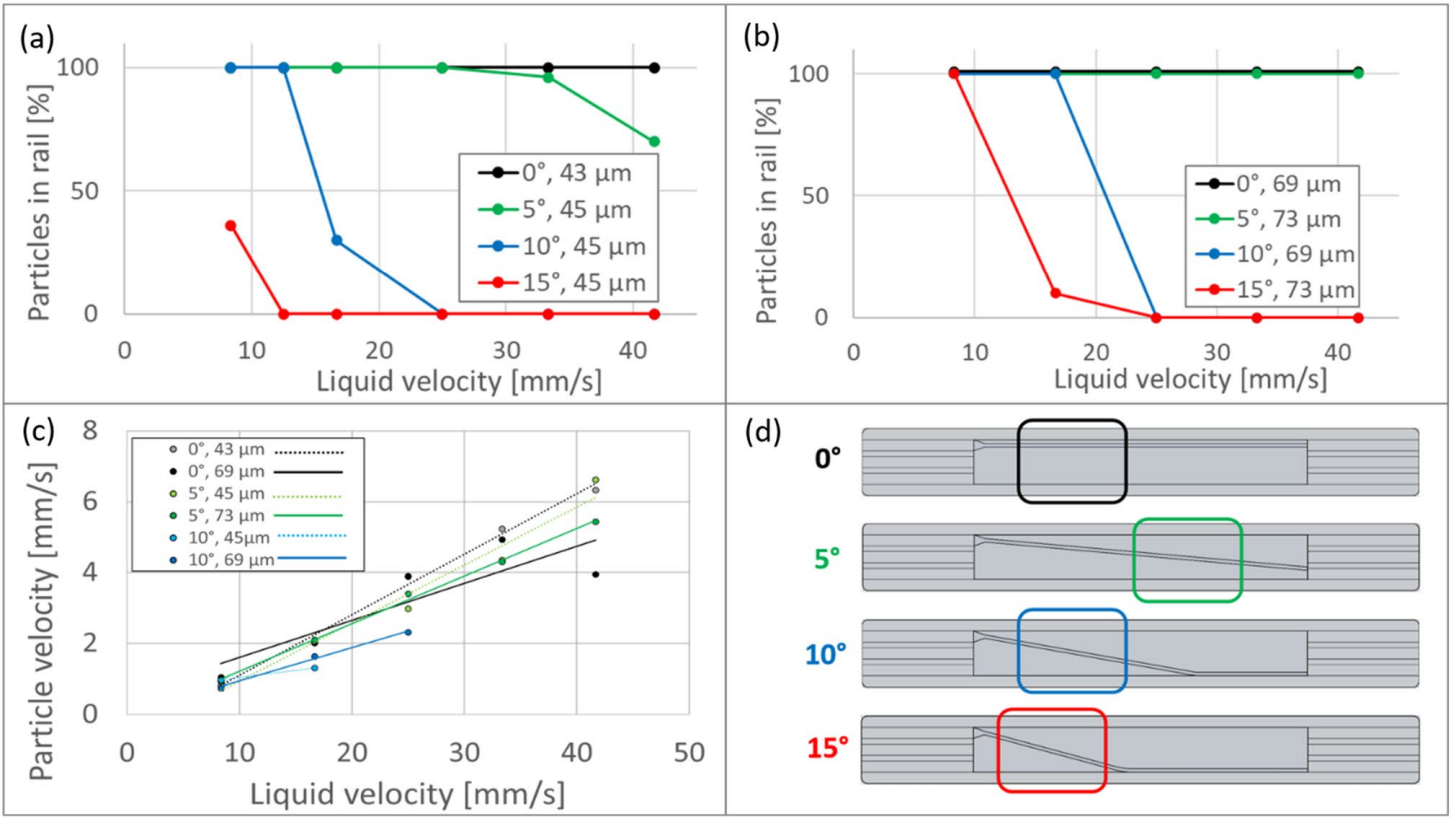
Fraction of the particles of ø 89 µm following the rail of depth (**a**) 45 ± 1 µm and (**b**) 72 ± 1 µm. (**c**) Velocity of particles following a rail. Stability and velocity of particles depends on rail depth, angle, and flow rate of the carrier liquid. (**d**) Scheme of the chips.

The behavior of the particles on the rails with the following angles to the channel axis was studied: 0°, 5°, 10° and 15° (see Fig. 6). The scheme of the chips is presented in Fig. 6d. The depths of the rail were 45 ± 1 µm and 70 ± 1 µm. The range of the liquid flow rate studied was 4.2–42.0 mm s^−1^. It was observed that at a linear liquid velocity below 6.5 mm s^−1^ particles do not travel undisturbed all the way through the chip and often stop to the surface of the chip.

We recorded the particles traveling in the rail and measured their velocity using GDPTlab v1.2 a Matlab GUI^31^. First, the colors of black and white pictures were inverted using a Matlab code because GDPTlab works only with dark field images (dark background and bright particle images). Then, we analyzed positions of each particle with GDPTlab and measured its displacement for a given time (Fig. 6c). Each point represents measurement on minimum 10 particles. The relative standard deviation (RSD) of the particle velocity was 15%. The liquid average velocity was calculated by measuring the time of collecting the liquid at the outlet of the chip in a measuring cylinder. The RSD of the liquid velocity was 5%. The velocity of the particle following the rail is much lower than that of the particle outside the rail. Overall, the particle displacement in the rail (in the rail direction) was about ten times slower than the average liquid velocity (in axial direction) in the channel (see the “Methods”, “Fluid velocity profile”).

**Figure 7:**
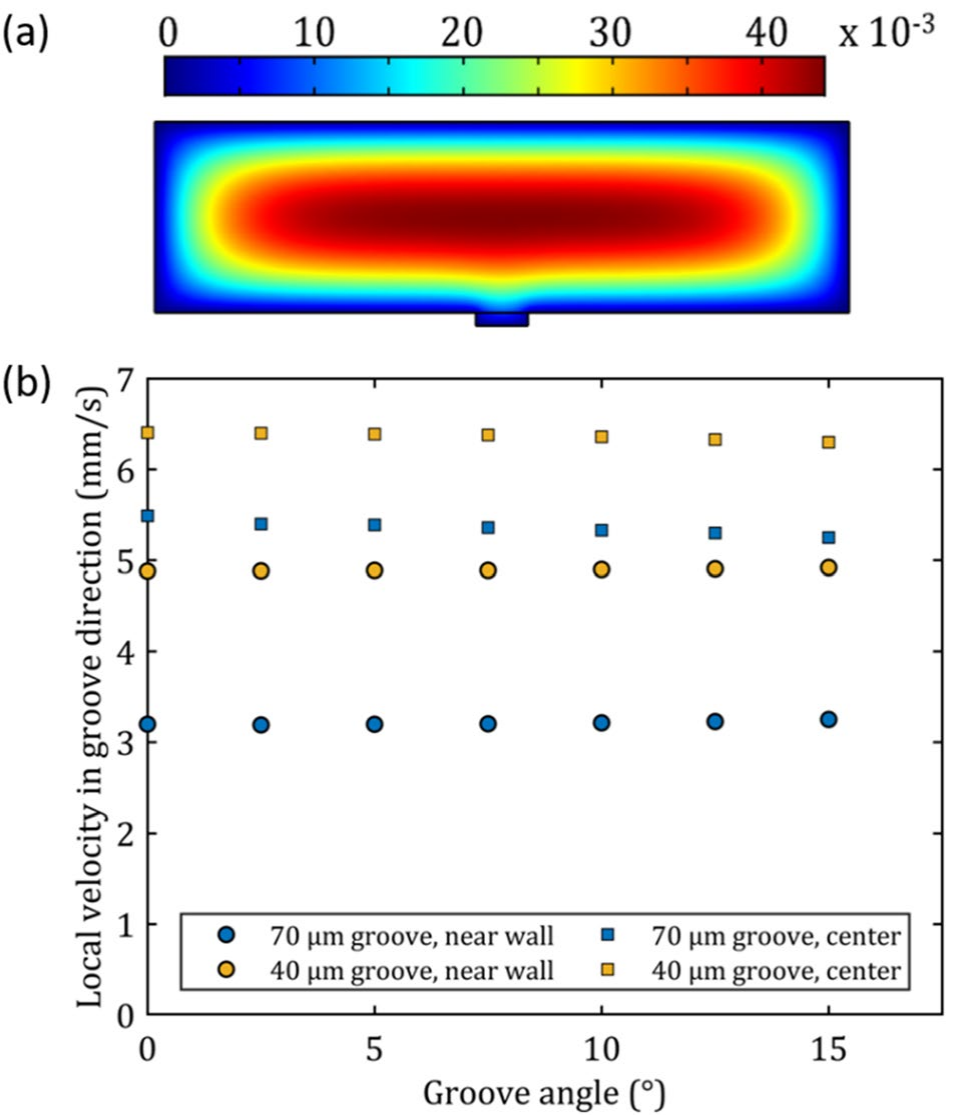
COMSOL simulation at a liquid flow rate of 25 mm s^−1^. (**a**) Cross section of the microfluidic channel showing the axial velocity field. (**b**) Local liquid velocity in the direction of the groove for different groove angles and depth, in the center of groove (squares) and close to the groove wall (circles).

The observed reduction in linear velocity can be attributed to a lower local velocity than the average velocity in the entire channel, on the one hand, and the occurrence of the (rotational and frictional) forces acting on the particle, on the other hand. To assess the magnitude of the velocity effect, COMSOL simulations were performed at different groove angles from 0° to 15°, assuming a fixed average axial flow rate of 25 mm s^−1^ Re = 18.4). The magnitude of the axial velocity field is shown in Fig. 7a. As can be noticed, the presence of the shallow groove has only a small influence on the axial velocity field in the microfluidic channel. To compare the observed velocity of the particles in the groove, the local liquid velocity in the groove direction at a height of the particle radius (44.5 µm) was measured. From Fig. 7b, it can be seen that slightly higher velocities are observed for a shallower groove. Moreover, the velocity decreases slightly with increasing angle. At 25 mm s^−1^, the liquid velocity at the level of the particle center is (5.4 ± 0.1 mm s^−1^ for 70 µm deep groove and 6.3 ± 0.1 mm s^−1^ for 40 µm deep groove) much smaller than the average flow velocity (25 mm s^−1^), but still considerable larger than the observed particle velocity (3.0 ± 1.0 mm s^−1^). The local velocity might vary slightly depending on the position of the rail. It was assumed that the particle remains near the center of the groove while, in reality, it is pushed towards the groove wall, where the velocity is slightly lower (i.e., 3.2 ± 0.1 mm s^−1^ for the 70 µm deep groove and 4.9 ± 0.1 mm s^−1^ for the 40 µm deep groove). The remaining velocity difference can be attributed to frictional and rotational forces (see section below for more details).

Figure 6a shows the fraction of the particles that follow the rail of 45 ± 1 µm depth. It is observed that as the velocity of the liquid increases, more particles escape the rail. It is notable that the angle of the rail is also an important factor. All particles travel within the trajectory of the rail of 0° and 5° until a liquid velocity of 25 mm s^−1^. As a comparison, for the same liquid velocity of 25 mm s^−1^, none of the particles remain in the rail of angles 10° and 15°. The larger the angle of the rail, the higher the fraction of the particles that escapes for a given liquid velocity. Another important factor determining particle stability is the depth of the rail. The fraction of the particles guided by the rail is much larger for the same liquid velocity and the rail angle condition when the rail is deeper (Fig. 6a,b).

The definition quality of the rail obviously also plays a critical role. The CNC (computer numerical control) machined rails (see “Used materials and chemicals”) have small imperfections. The local roughness (expressed as *R*_*g*_) determined by a profilometer (Filmetrics Profilm 3D) was always in the sub-micron range (see Fig. 2b for a characteristic roughness plot) for all tested channels. Differences in channel depth along a single channel as measured on five different locations were significantly larger and reached up to 7 µm between the maximal values (highest and lowest point) within a single channel. Chip to chip variance in depth expressed a standard deviation was 1.0 µm and 2.3 µm for 43.8 µm and 71.0 µm deep channels, respectively (*n* = 4).

Although the lines representing the fraction of the particles in the rail as a function of liquid velocity for similar chips are not identical, it is remarkable that they all have the same position of the threshold at which the particles start to be unstable in the rail and escape.

Imperfections of the surface of the lateral walls and the bottom of the rail may trigger the escape events. Indeed, if the surfaces of the rail were perfect, then whether a particle escapes or not from the rail would be determined by the balance of the forces. For example, in a deep enough rail particles would remain inside the rail as long as the lift forces are insufficient to cause the escape, and this would hold for the entire length of the rail. On the other hand, if the balance of the forces is opposite such as, e.g., in a very shallow rail, then the particles would escape from the rail. However, as observed in the experiment, when the angle of the rail with respect to the direction of the flow increases, the escape events start to occur with increasing rate at different, i.e., random, positions of the rail: sometimes particles escape at the very beginning of the rail and sometimes this happens at different positions, or does not happen at all (i.e. the particle remains inside the rail). This behavior implies that the process of escape can be modelled by adding a random force in the equations of motion, similarly to the thermal force in the case of Brownian particles. This random force can be interpreted as caused by random imperfections, or roughness, at the surface of the rail.

**Figure 8:**
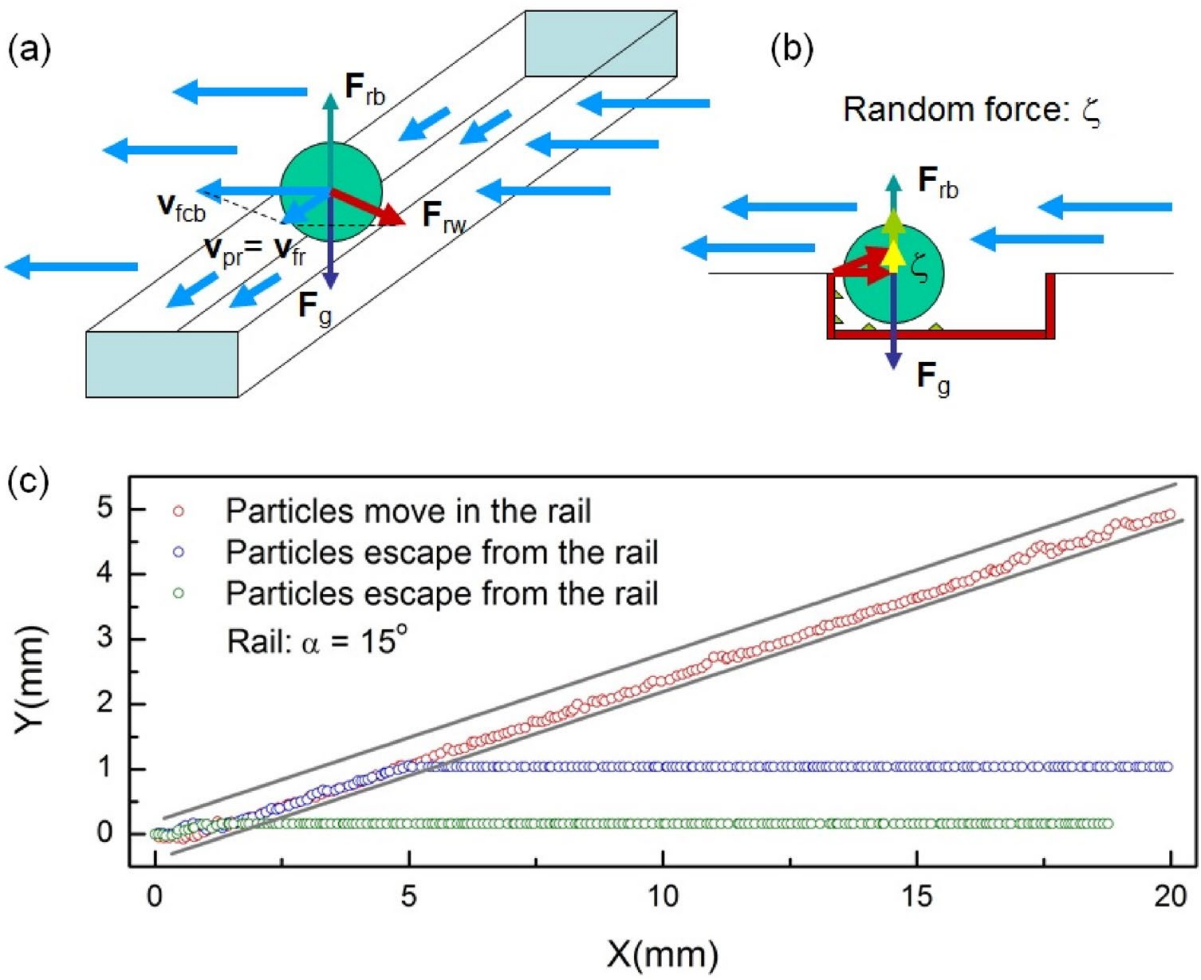
(**a**) The motion of a particle, driven by the fluid flow, in the rail. Long blue arrows show the fluid flow in the chip, and short blue arrows indicate the flow in the rail. Main forces and velocities (in the overdamped regime): the gravity force, *F*_g_, is compensated by the reaction force from the bottom of the rail *F*_rb_, the particle velocity in the rail, *v*_pr_, proportional to the fluid velocity in the rail resulting from the fluid velocity at the bottom of the chip, *v*_fcb_, and the wall reaction force *F*_rw_. (**b**) Imperfections at the bottom and lateral walls of the rail are modeled by a random force. (**c**) Examples of simulated particle trajectories: particles escape from the rail (blue and green circles) and following the rail (red circles).

In general, the motion of a particle driven in a rail by fluid flowing along the channel in the setup shown in Fig. 8, obeys the following equation of motion:

1 $$m\ddot{r}={F}_{g}+{F}_{b}+{F}_{l}+{F}_{dr}+{F}_{w}+{F}_{d}+{F}_{wf},$$

where *m* is the mass of the particle, $\ddot{r}$ denotes the second derivative of the coordinate *r* with respect to time *t*, and the forces ${F}_{g},{F}_{b},{F}_{l},{F}_{dr},{F}_{w},{F}_{d}$ and ${F}_{wf}$ stand for the gravity, buoyancy, the lift force(s), the driving force, the wall reaction force, the Stokes drag and the wall friction forces, respectively.

As observed in the experiment, particles move with a constant velocity, along nearly straight trajectories inside the rail, which means that the governing forces in Eq. (1) balance each other, and the motion of particles is overdamped. This allows us to considerably simplify the description.

Thus, the particles move with a constant velocity proportional to the fluid velocity at the level of the particle (see “Methods”, “Fluid velocity profile”). In addition, their motion is affected by a random force due to the imperfections. This motion can be modelled by simple Langevin-type overdamped equations in two dimensions,

2 $$\begin{array}{c}\dot{x}={v}_{0}\mathrm{cos}\theta +{\xi }_{0, x}\left(t\right)\\ \dot{y}={v}_{0}\mathrm{sin}\theta +{\xi }_{0, y}\left(t\right)\end{array} ,$$

where *v*_0_ is the velocity of the particle driven by the fluid flow (in the absence of other forces), *θ* is the direction of the flow with respect to the chip channel, ${\xi }_{0}\left(t\right)=({\xi }_{0, x}\left(t\right),{\xi }_{0, y}\left(t\right))$ is a two-dimensional non-correlated thermal-like Gaussian noise (due to the imperfections) with correlation functions: $\langle {\xi }_{0,i}\left(t\right)\rangle =0$ and $\langle {\xi }_{0,i}\left(t\right){\xi }_{0,j}\left(0\right)\rangle =2{D}_{0}{\delta }_{ij}\delta \left(t\right)$, where (*i*, *j*) = (*x*, *y*), *D*_0_ is the diffusion coefficient, and δ_*ij*_ and δ(*t*) are the Kronecker delta symbol and delta-function, respectively. Note that Eq. (2) is similar to the Langevin equations describing the motion of driven or self-propelled particles^32–35^, where the driving velocity *v*_0_ corresponds to self-driven velocity of self-propelled particles. The random force ${\xi }_{0}\left(t\right)$ in Eq. (2) is Gaussian distributed, and the measure of its average amplitude is the “effective temperature”, *T*_eff_, which in general case is the “noise intensity”^36^, or a measure of fluctuations^35^. In case of the Brownian motion, *T*_eff_ is temperature *T*, which is related to the diffusion coefficient, *D*_0_, via Stokes–Einstein formula: *D*_0_ = *k*_B_*T*/(6πη*r*_p_), where *k*_B_ is the Boltzmann constant, η is the fluid viscosity, and *r*_p_ is the radius of the particle. In general case, *T*_eff_ is a measure of fluctuations, not only thermal fluctuations, but of any nature, e.g., fluctuations of the position (e.g. due to the roughness of the rail surface).

The effect of roughness of the channel walls can be introduced via the renormalization of the effective diffusion coefficient, *D*_eff_, as follows. When a particle executes random walk, characterized by persistence time τ_rw,_ and velocity *v*_rw_, the corresponding *D*_eff_ can be presented as *D*_eff_ = *D*_0_ + *D*_rw_, where *D*_rw_ = τ_rw_(*v*_rw_)^2^/4 is the contribution due to the random walk^33,37^. The effect of roughness results in random movements of a particle with velocity *v*_rg_, which is of the order of the driving velocity of the flow, i.e., *v*_rg_ ~ *v*_flow ∙_ cos(θ), and with persistence time τ_rg_ = *R*_g_/*v*_rg_, where *R*_g_ characterizes spatial roughness of the walls. Therefore, we can approximate *D*_eff_ as follows: *D*_eff_ = *D*_0_ + *R*_g_*v*_rg_/4. Note that for large enough particles of *r*_p_ ~ 100 μm and sub-micrometer roughness of the channel walls, *R*_g_ ~ 0.1 μm, *R*_g_*v*_rg_/4 >  > *D*_0_, and therefore *D*_eff_ is determined by the roughness of the channel walls, *D*_eff_ ≈ *R*_g_*v*_rg_/4, while thermal diffusion is negligibly small in comparison to the random movements due to the wall imperfections. Using Stokes–Einstein formula, we can formally introduce an effective “temperature”, *T*_eff_, that characterizes the fluctuations of the particle motion due to the roughness of the channel wall, *D*_eff_ = *k*_B_*T*_eff_/(6πη*r*_p_). We note that this quantity, *T*_eff_, has a different nature than usual *T* (which is a measure of thermal noise) and characterizes the intensity of the noise resulting from the collisions of a particle with the surface roughness: *T*_eff_ = *f*(*R*_g_).

The simulation results for the angle *θ* = 15° and *R*_g_ ~ 0.1 µm for an 89 µm particle are presented in Fig. 8, where three typical trajectories are shown. One trajectory corresponds to the case when the (random) escape event occurs at the very beginning of the motion of a particle in the rail. The other one shows an escape around the middle of the rail. After the escape, the particles move on the bottom of the chip following the direction of the fluid. Finally, the case is shown in the figure when a particle does not escape and remains in the rail. The presented simulated trajectories are similar to those observed in the experiment. Note that all the trajectories shown in Fig. 8 are calculated for the same set of parameters, and the escape events are determined by a random force modelling the channel wall imperfections.

#### Tests on zig zag chip

**Figure 9:**
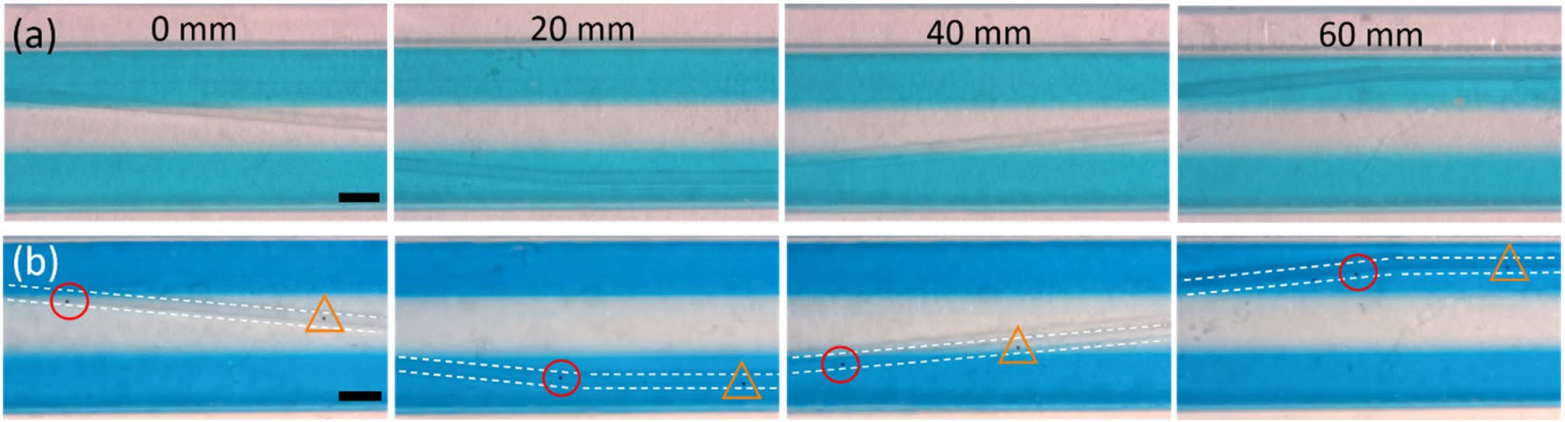
(**a**) Optical microscopy picture of (**a**) the channel at different positions in the chip; (**b**) the particles following the rail in the first zig-zag. Side liquids: EtOH with blue dye, middle liquid: EtOH. All three liquids are introduced at liquid velocity of 25 mm s^−1^. Scale bar is 1 mm.

After defining the optimum geometry of the rail and range of the liquid velocity, the conditions as described above were applied to a zig-zag chip, see Fig. 1d. The zig-zag chip has a rail of 70 ± 1 µm built of alternating rails with angles: −5°, 0°, 5°, 0° (first zig-zag) and again −5°, 0°, 5°, 0° (second zig-zag). The first three streams of ethanol were introduced at a velocity of 25 mm s^−1^. Side streams were colored with Patent Blue for the visualization of the liquid flow. The particles are introduced in the middle (ethanol) stream. The side liquid streams contain blue colored ethanol. Through the entire length of the zig-zag chip, the liquid behavior follows Regime 1. This can be seen in Fig. 9a and Fig. 2S. Moreover, diffusion of the dye from the side streams to the middle stream is negligible for the entire chip length.

Particles were introduced in the chip through the middle stream. This gives them the possibility to be trapped in the rail while they are still present in the rinsing solution. This guarantees that all particles spend the same time in the side stream. Particles were following along the rail that is presented in Fig. 9b, the first zig-zag, and Fig. 3S (the first and second zig-zag). The distance between particles changes depends on the position in the chip. Closer to the side (rail 0°) the particles move slightly slower and approach closer to each other. Therefore, it is preferred that particles are introduced in the chip with a distance of > 5 mm between them.

### Deposition of polyelectrolyte bilayer(s) on PMMA-MAG-NH2

**Figure 10:**
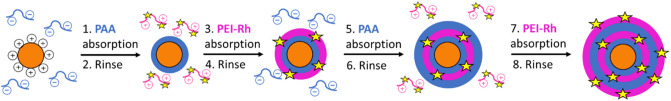
Schematic representation of a PMMA-NH2 particle deposited with poly (acrylic acid) (PAA) and poly (ethylenimine) labeled with Rhodamine (PEI-Rh) bilayers by the layer-by-layer (LbL) technique. Steps 1–4 show the deposition of one bilayer of PAA/PEI-Rh (LbL-1-PMMA-NH2) on a PMMA-NH2 particle. Steps 5–8 show the deposition of second bilayer of PAA/PEI-Rh (LbL-2-PMMA-NH2) on a particle.

A solution of poly (acrylic acid) PAA (0.033% w/w) in ethanol and a solution of poly(ethylenimine) labeled with rhodamine PEI -Rh (0.033% w/w) in ethanol were used. Each step of coating was alternated by rinsing the particles with ethanol (Fig. 10). The deposition of the PAA/ PEI-Rh bilayer was verified using fluorescence microscopy.

#### Batch experiment

For deposition of the first layer, 0.5 mL of a PAA solution was added to a glass vial containing positively charged PMMA-MAG-NH2. Adsorption was allowed to proceed for 10 min followed by gentle shaking. After that, particles were kept at the bottom of the vial with the help of a magnet, then the solution was removed, and the particles were washed twice with ethanol. A 0.5 mL of PEI-Rh solution was then added to the PAA coated particles and allowed to interact for 10 min, followed by removal of the solution and washing with ethanol. The process was repeated, leading to the deposition of a second PAA/PEI-Rh bilayer.

#### On chip experiment

**Figure 11:**
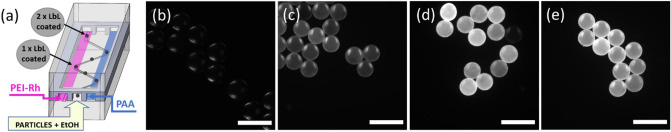
(**a**) Schematic representation of PMMA-NH2 particles deposited with poly (acrylic acid) (PAA) and poly (ethylenimine) labeled with Rhodamine (PEI-Rh) bilayers by the layer-by-layer (LbL) technique on chip. (**b–e**) Fluorescence photographs of PMMA-NH2 magnetic particles: (**b**) particles without coating (control), (**c**) particles with one bilayer (LbL-1-PMMA-NH2), coated in batch, (**d**) particles with two bilayers (LbL-2-PMMA-NH2), coated in batch, (**e**) with two bilayers (LbL-2-PMMA-NH2), coated on chip. Scale bars are 200 µm.

Next, coating of the particles was performed with the zig-zag chip, which has a rail of 75 ± 1 µm. The side streams are composed of PAA ethanol solution (polyanion solution) and PEI-Rh ethanol solution (polycation solution). Ethanol is introduced in the middle stream as a rinsing solution. Positively charged PMMA-MAG-NH2 particles (98.5 µm diameter) are introduced in the middle stream and are sequentially carried by the PAA solution, ethanol and PEI-Rh stream in order to undergo deposition of the first bilayer (the first zig-zag). After following the trajectory of the second zig-zag, the second bilayer is deposited, see Fig. 11a. Particles were collected at the outlet of the chip into a glass beaker containing ethanol. After the particles sedimented, the liquid was removed by washing the particles twice with ethanol. Fluorescent microscopy pictures confirmed presence of two bilayers, see Fig. 11e. The fluorescence intensity was comparable with that of the particles with two bilayers coated in batch.

On Fig. 11b–e, fluorescent microscopy images are presented of PMMA-MAG-NH2 particles with (b) zero, (c) one, and (d) two bilayers. Particles without coating show no fluorescence. Particles with two bilayers show a higher intensity than particles with one bilayer.

The whole process of coating the particles with four sublayers required seven sequential steps: 1-PAA, 2-washing, 3-PEI-Rh, 4-washing, 5-PAA, 6-washing, 7-PEI-Rh and took about 1 min.

It is worth to mention that the time particles were exposed to each of the coating solutions and that the washing solution was short (a dozen of seconds), which was sufficient to undergo the deposition of a sublayer. It is possible that the fact that the liquid is continuously refreshed inside the rail, and particles both slide and rotate while moving, helps for the efficient particles coating. This is again to advantage to perform LbL on chip.

The LbL coating is usually quick but in case when a longer time of reaction is needed, the flow rates of liquids introduced can be decreased. This must be carefully done in such a way that the system is still in Regime 1 and not in Regime 3. In order to remain in Regime 1, it is possible to change geometry of the chip: e.g., elongate or widen the channel, or eventually introduce walls between streams. Our system can be applied to particles of different diameters. This will require adaptation of the depth of the rail. A careful estimation of the optimal dimension will be the subject of our next study. Thanks to the fact that no external force is applied, our system is universal for particles and potentially for heavy or floating droplets (inverting the chip in the latter case). Relatively high concentration of particles can be processed in continuous way.

Our system can be used in multiple chemical and biological assays (for example, immunoassays) that require numerous liquid reagents and washes that are introduced sequentially. We see the advantage of using the engraved rail that our system is less prone to clogging than guiding structures that obstruct the flow and the particles it carries, resulting in a filtering action.

## Materials and methods

### Used materials and chemicals

PMMA chips were designed and fabricated inhouse with the use of high-speed CNC milling machine (Datron Neo, Datron AG., Germany). The depth of the grooves was measured with a profilometer (Filmetrics Profilm 3D). The parts of the chip were assembled and bonded with the use of ethyl acetate that was introduced in the discrete amount between the layers of the chip^38^. Glass capillaries (inner diameter (ID) 450 µm, outer diameter (OD) 670 µm, Polymicro, Achrom) were glued to the chip inlets and outlets in order to introduce the liquids with the use of syringe pumps or pressurized pumps (Fluigent). In For the particle suspension, a vortex mixer (VWR) was used to vibrate the Falcon tube containing the particle suspension to prevent sedimentation of particles at the bottom of the Falcon tube.

In our work we used magnetic polystyrene particle (PS-MAG-AR110, 89 µm, SD = 1.2 µm, Iron oxide = 10%, Microparticles GmbH) and magnetic amino functionalized poly(methyl methacrylate)particles (PMMA-MAG-NH2, 98.5 µm, Microparticles GmbH). Magnetic properties of the particles are not required for our experiments but at the stage of optimizing the setup were convenient for regenerating the device.

Experiments were performed in technical grade ethanol. Patent blue (Aldrich) was used as a colorant to visualize the flows in chip.

Polyethyleneimine, (PEI, branched, average 25 kDa by LS average Mn 10 kDa by GPC) and Poly (acrylic acid) (PAA) (35 wt; % solution in water, typical MW 100 kDa) were purchased from Aldrich. Rhodamine isothiocyanate was purchased from Cayman Chemical Company.

### Labeling of PEI with rhodamine

PEI was dissolved in dimethyl sulfoxide, DMSO (Sigma-Aldrich) together with rhodamine B isothiocyanate (RITC, mixed isomers, Cayman Chemical Company). Mixture was stirred for 5 h. After that ethanol was added to dilute the PEI to 1%. Mixture was dialyzed against ethanol (dialysis bag with cut off 12–15 kDa) for one week in order to remove DMSO and not reacted RITC. Concentration of dialyzed PEI-Rhodamine (PEI-Rh) labeled solution was calculated as 0.87% and used as a stock solution.

### Fluid velocity profile

As shown by J. Pazourek and J. Chmelik^39^, the fluid velocity in a channel with a rectangular cross-section where one side is much larger than the other one, *h* >  > *w*, is related to the coordinate *y* by the simple analytical expression:

3 $$v\left(y\right)=\frac{\Delta p }{2\mu L}.\left( \frac{{ w}^{2}}{4}- {y}^{2}\right),$$

where $\Delta p$ is the pressure drop between the opposite sides of the channel, $\mu$ is the dynamic viscosity of the fluid, *L* is the length of the channel, and *w* is the height of the channel in the *y*-direction.

The liquid velocity can be calculated directly from Eq. (3). However, we know the average velocity of the liquid, <*v*>, measured experimentally, and therefore it is useful to express *v*(*y*) via this known quantity. The average velocity can be calculated by integrating Eq. (3) along the height*,* from −*w*/2 to *w*/2, and dividing by *w*, resulting in:

4 $$<v>=\frac{\Delta p}{2\mu L}\frac{{ w}^{2}}{6}$$

and

5 $$v\left(y\right)=\frac{6<v>}{{w}^{2}}\left( \frac{{ w}^{2}}{4}- {y}^{2}\right).$$

This result, Eq. (5), is valid for a channel with a rectangular cross-section where the width is much larger than the height, *h* >> *w*, and the maximum value,

6 $${v}_{max}= \frac{3}{2}<v>$$

is achieved along the line *y* = 0.

The chip has a rectangular cross-section with comparable width and height, 4 mm × 1 mm. Therefore, it is reasonable to assume that the fluid velocity profile is parabolic in both directions, in the *x*- (width) and *y*-direction (height), and the maximum velocity is achieved at one point (*x* = 0, *y* = 0). This means that *v*_max_ in Eq. (6) becomes a function of *x*, and its average, <*v*_max_(*x*)>, is related with *v*_max_(*x* = 0) via the same relation as <*v*> and *v*_max_ in Eq. (6):

7 $${v}_{max}= \frac{3}{2}<{v}_{max}\left(x\right)> = \frac{9}{4}<v>.$$

Thus, the average velocity in a channel with parabolic velocity profile in both directions is 2/3 of its value for a channel with infinite width [Eqs. (3)–(5)]. Therefore, in order to evaluate fluid velocity *v*(*y*) for x ≈ 0, the right-hand side of Eq. (5) should be corrected by a factor of 3/2.

Thus, for a particle of diameter of 89 μm, the fluid velocity at the level of the center of the particle is approximately

8 $$v\left(y=\frac{w}{2}-r\right)=0.2<v>.$$

This analytical result is also consistent with the estimate of the fluid velocity profile found from COMSOL simulations.

Inside the rail, the flow can be approximately considered as a Couette flow, being top layer driven by the flow near the bottom of the chip and having zero velocity at the bottom of the rail. Therefore, the fluid velocity further decreases in the rail, and for a particle 89 μm in a rail of about 100 µm deep, the velocity is estimated as ≈ 0.1 of the average value in the chip.

## Conclusions and future perspectives

Particle manufacturing, coating or biologic essays are laborious and time demanding procedures, which require that reacting and/or washing solutions are loaded separately and sequentially one at the time.

In this paper, we present a novel, passive method to guide high concentrations of particles in microfluidic channels. Moreover, we have shown that Layer-by-Layer coating of particles has been successfully performed on chip using a rail guided method. We automated a LbL coating procedure into one continuous process and demonstrated that the chip can replace seven consecutives steps in batch. The use of a rail provides control over the trajectory of the particles and ensures that all particles follow the same route. The depth and angle of the rails together with the liquid velocity were studied to provide a suitable channel geometry.

Moreover, we have shown that when laminar co-flows are introduced into the channel that contains a groove at the bottom, with nonzero angle, three different types of behavior of liquid (regimes) are observed, determined by the groove depth and liquid velocity.

We demonstrated the functionality of our device by on chip coating of particles with two bilayers (four sublayers), which was confirmed by fluorescence microscopy. All steps, that in batch require multiple manipulations of particles (seven consecutive steps), were taken in a single device, which took about one minute only.

This paves the way for a wide range of applications for which multilayers can be applied in a single continuous process. When much larger layer numbers are needed, diffusion between the streams will limit the residence time in the channels, and as a result the attainable layers, because the different coating and washing streams will eventually merge in a single (partially) mixed stream. In a future study, we will integrate obstruction structures to minimize diffusion between the layers, allowing to increase the residence time, which will enable the application of tens of layers. This will allow for the most demanding optical applications wherein large layer numbers with well-defined layer thicknesses are critical. Tailored particles for controlled drug delivery with advanced release trigger mechanisms are another appealing prospect for the concept introduced in the present paper.

While in the present paper PMMA was used as channel substrate, it is less chemically resistant than the commonly used glass substrate in the chemical industry. To be operated in chemically harsh conditions, alternative formats as patterned glass (or silicon on which an oxide layer has been applied as a last step) can e.g. be used, with the grooves obtained by deep reactive ion etching (resulting in very vertical walls). This will allow to maintain variation on both short and long-range scale in the sub-micron region, which is favorable for a further increase in particle stability, hence allowing for more flexibility in terms of flow rate and control of residence time in the streams.

## Supplementary Information

Supplementary Figures.

## Data Availability

The data generated and analyzed during this study are included in this published article and in the supplementary information files.
