# Supplementary material for: Rail induced lateral migration of particles across intact co-flowing liquids
Source: Sci Rep. 2022 Dec 16;12:21775. doi: 10.1038/s41598-022-26387-5 (PMC9758194; doi:10.1038/s41598-022-26387-5)
Supplement: Supplementary file 1 — Supplementary Figures. [file 41598_2022_26387_MOESM1_ESM.docx]

**Supplementary Information**

**Rail induced lateral migration of particles across intact co-flowing liquids**

Iwona Ziemecka,^a^ Amaury de Hemptinne,^a^ Vyacheslav R. Misko,^a^ Matthieu Briet,^a^ Pierre Gelin,^a^ Ilyesse Bihi,^a^ Dominique Maes,^b^ and Wim De Malsche,^a*^

^a^ µFlow group, Department of Chemical Engineering Vrije Universiteit Brussel, Pleinlaan 2, 1050 Brussels, Belgium

^b^ Structural Biology Brussels, Vrije Universiteit Brussel, Pleinlaan 2, 1050 Brussel, Belgium

Below, supplementary figures mentioned in the main text are presented: Figures 1S, 2S and 3S.


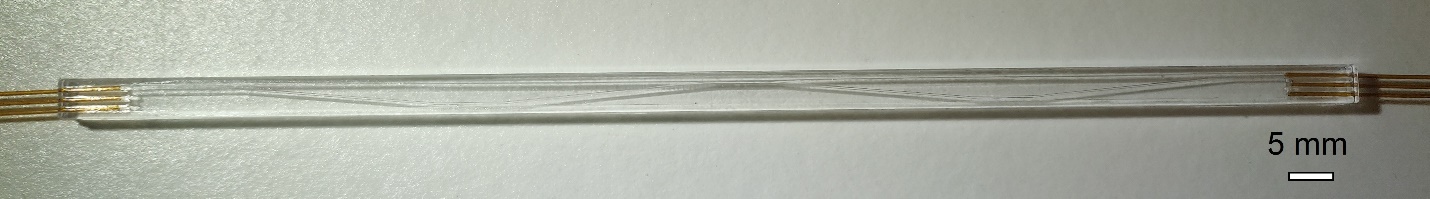


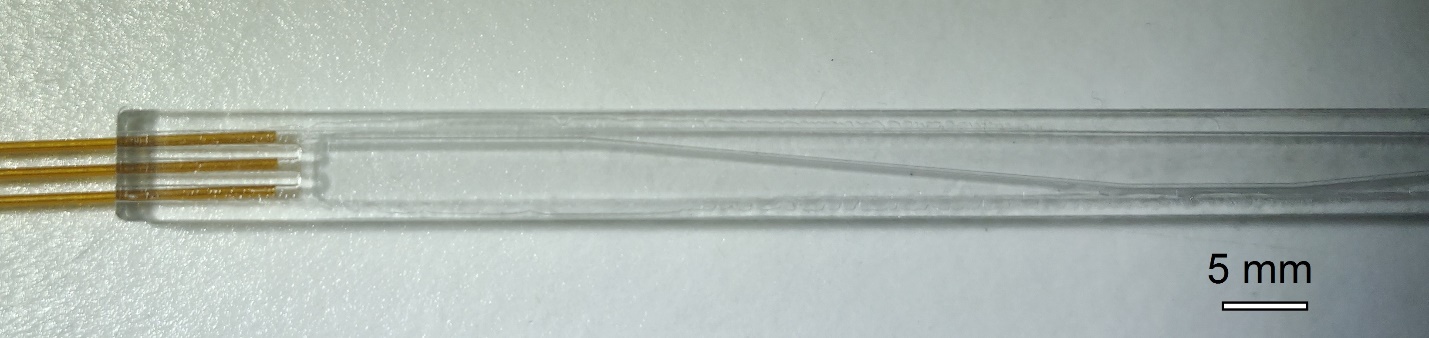


**Figure 1S**. A PMMA chip with a zig-zag channel. Top panel: an image of the chip; bottom panel: a zoom image of the same chip. The channel width is 4 mm.


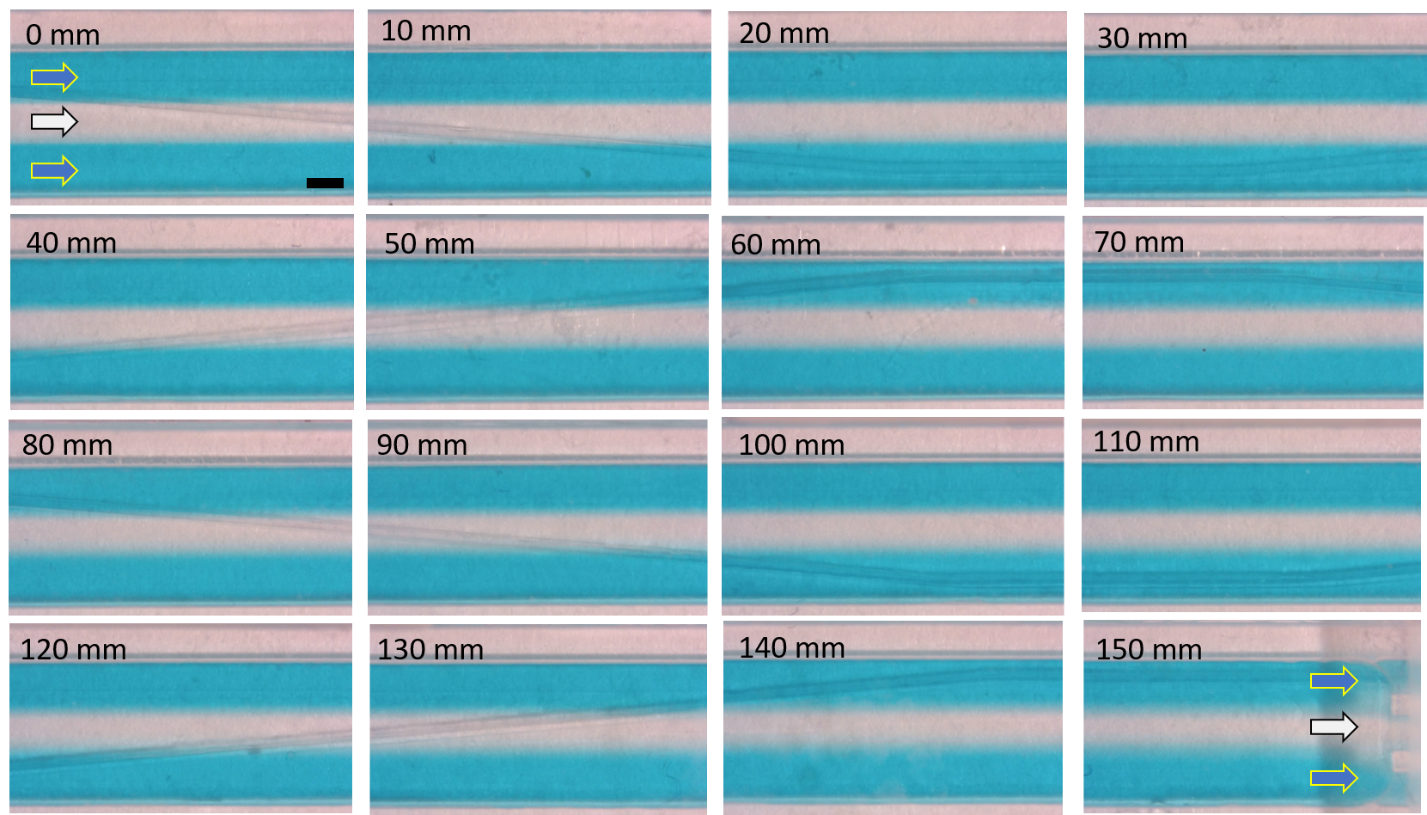


**Figure 2S.** Optical microscopy pictures of the channel at the different position of the chip. Side liquids: EtOH with blue dye, middle liquid: EtOH. All three liquids are introduced at liquid velocity of 25 mm s^-1^. The scale bar is 1 mm. The radial diffusion of the blue dye over the length of the chip (15 cm) is negligible.


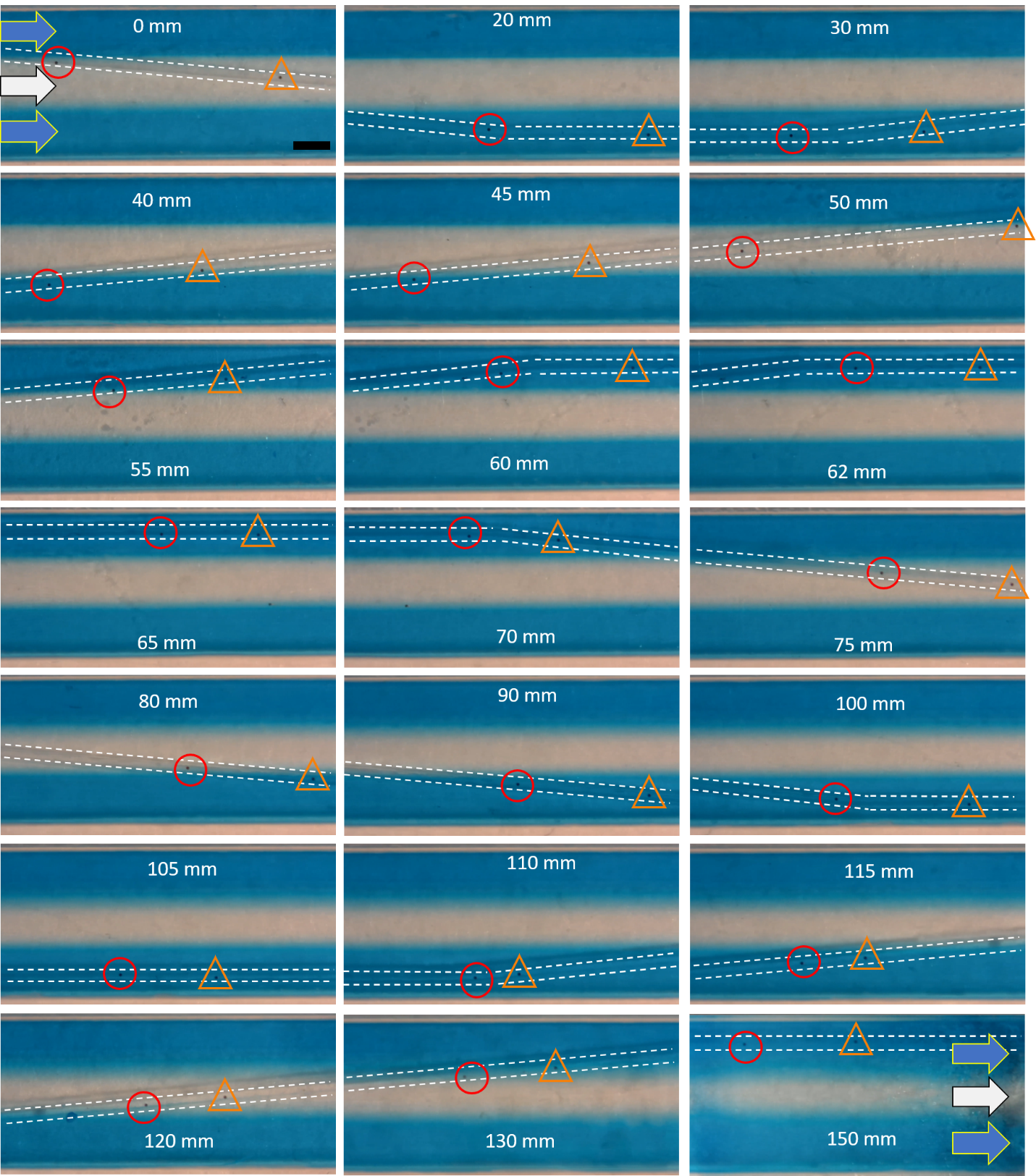


**Figure 3S.** Optical microscopy pictures of two particles traveling along the rail at different position of the chip. Full trajectory. Side liquids: EtOH with blue dye, middle liquid: EtOH. Liquid velocity is 25 mm s^-1^. The scale bar is 1 mm.
